# Use of the Food and Nutrition Surveillance System in epidemiological studies on the nutrition profile of the Brazilian population: a scoping review, Brazil, 2008-2025

**DOI:** 10.1590/S2237-96222026v35e20251036.en

**Published:** 2026-07-26

**Authors:** Caroline de Oliveira Gallo, Luiza Navarro de Azevedo, Patricia Constante Jaime

**Affiliations:** 1 Universidade de São Paulo, Departamento de Nutrição, São Paulo, SP, Brazil Universidade de São Paulo Departamento de Nutrição São Paulo SP Brazil; 2 Universidade de São Paulo, Núcleo de Pesquisas Epidemiológicas em Nutrição e Saúde, São Paulo, SP, Brazil Universidade de São Paulo Núcleo de Pesquisas Epidemiológicas em Nutrição e Saúde São Paulo SP Brazil

**Keywords:** Food and Nutritional Surveillance, Health Information Systems, Nutritional Status, Nutritional Epidemiology, Scoping Review, Vigilancia Alimentaria y Nutricional, Sistemas de Información en Salud, Estado Nutricional, Epidemiología Nutricional, Revisión de Alcance

## Abstract

**Objectives:**

To identify and characterize the scientific literature on the use of the Food and Nutrition Surveillance System (SISVAN) in the description and analysis of the nutrition profile of the Brazilian population who use the Brazilian Unified Health System.

**Methods:**

This is a scoping review. Document selection occurred independently in stages previously defined in a research protocol. The review included primary studies, reviews, and grey literature that underwent peer review and produced evidence from quantitative information on nutrition status held on SISVAN Web, for all populations and locations. Data extraction was standardized, and publications were analyzed according to a glossary developed to standardize methodological aspects.

**Results:**

A total of 1,294 records were identified, and 161 were analyzed after applying the eligibility criteria. There was a significant increase in publications, especially from 2021 onwards, with emphasis on ecological time-series designs, which have adopted robust methods to analyze SISVAN data. Twenty-nine studies were identified that worked with individualized data, with cross-sectional or retrospective cohort analysis. The most studied life stage was childhood, particularly up to 5 years of age. In 40 studies, the data source was stratified, and most used data from the *Bolsa Família* Program. Pernambuco, Minas Gerais and Bahia were the states most studied. Some studies investigated the effect of the COVID-19 pandemic on SISVAN indicators or used georeferencing/spatial analysis methods.

**Conclusion:**

This review presented the state of the art of scientific production using nutrition information from SISVAN and characterized methodological aspects, thus informing future research.

## Resumen

**Objetivos:**

Identificar y caracterizar la literatura científica sobre el uso del Sistema de Vigilancia de Alimentos y Nutrición (SISVAN) en la descripción y el análisis del perfil nutricional de la población brasileña que utiliza el Sistema Único de Salud.

**Métodos:**

Se trata de una revisión de alcance. La selección de documentos se realizó de forma independiente en etapas previamente definidas en un protocolo de investigación. Se incluyeron estudios primarios, revisiones y literatura gris que fueron sometidos a revisión por pares y que aportaron evidencia a partir de información cuantitativa sobre el estado nutricional del SISVAN Web, para todas las poblaciones y localidades. La extracción de datos se estandarizó y las publicaciones se analizaron según un glosario desarrollado para estandarizar los aspectos metodológicos.

**Resultados:**

Se identificaron 1294 registros y se analizaron 161 tras aplicar los criterios de elegibilidad. Se observó un incremento significativo en las publicaciones, especialmente a partir de 2021, destacando los diseños de series temporales ecológicas, que han adoptado métodos robustos para analizar los datos del SISVAN. Se identificaron 29 estudios que trabajaron con datos individualizados, con análisis de cohortes transversales o históricas. La etapa de vida más estudiada fue la infancia, particularmente hasta los 5 años de edad. En 40 estudios, la fuente de datos fue estratificada, y la mayoría utilizó datos del Programa *Bolsa Família*. Pernambuco, Minas Gerais y Bahía fueron los estados más estudiados. Algunos estudios investigaron el efecto de la pandemia de COVID-19 en los indicadores del SISVAN o utilizaron métodos de georreferenciación/análisis espacial. **Conclusión:** Esta revisión presentó el estado del arte de la producción científica utilizando información nutricional del SISVAN y caracterizó aspectos metodológicos, lo que permite orientar futuras investigaciones.

## Introduction 

The Brazilian National Food and Nutrition Policy recognizes food and nutrition surveillance as a means of qualifying care in the Brazilian Unified Health System (*Sistema Único de Saúde*, SUS)^1^. To this end, the Food and Nutrition Surveillance System (*Sistema de Vigilância Alimentar e Nutricional*, SISVAN) acts to enable diagnosis of the food and nutrition status of the population and identifies geographic areas and groups at risk, supporting SUS care provision and public health decisions^2^.

Food and nutrition surveillance practices occur mainly in Primary Health Care (PHC). Based on anthropometric and food consumption assessments, and screening for Food and Nutrition Security, those practices are operationalized by the SISVAN Web health information system, created in 2007 to centralize information management^3^^-^^5^. This platform receives data from the national PHC central information hub (e-SUS PHC strategy)^6^, from the *Bolsa Família* Program (conditional cash transfer) Management System, as well as data input directly to the platform by SUS managers. The data can be accessed in publicly available reports, which contain frequencies of nutrition classifications in geographic areas or health establishments on an intramunicipal scale^5^, or accessed via a restricted module for Health Department managers and technicians, who access individualized data^7^.

Food and nutrition surveillance has been the subject of interest in scientific research in the field of public health nutrition. Researchers have used SISVAN data to support investigations into the food and nutrition profile of the population that uses the SUS, the performance of this system and the quality of the data collected^8^^-^^14^.

Despite the importance of SISVAN for generating evidence in the field of surveillance and the analytical potential of the continuously collected data, there are no studies that systematize the literature produced. It is therefore relevant to systematically map scientific production based on SISVAN, in order to understand the extent and type of evidence being produced and to identify gaps, strengths and key aspects in the knowledge about this information system.

The objectives of this review were to identify and characterize the scientific literature on use of SISVAN Web in the description and analysis of the nutrition profile of the Brazilian population. It seeks to understand how SISVAN Web information has been used in scientific production, identifying the most studied populations in terms of age group, location, sex, race/color and education level; the most frequently evaluated nutrition status indicators; and the most commonly used research designs and data analyses.

## Methods 

Design

This is a scoping review guided by the Joanna Briggs Institute manual and the checklist of the Preferred Reporting Items for Systematic Reviews and Meta-Analyses Extension for Scoping Reviews^15^^-^^17^, as well as by the population-concept-context elements:

Population: individuals served by SUS Primary Health Care with consolidated data on the SISVAN Web system, covering all phases/events of their life course, including gender, race/color and education level.

Concept: epidemiological studies on the diet and nutrition profile of the population with data consolidated on the SISVAN Web system.

Context: SUS Primary Health Care, covering Brazil as a whole, in all its states and municipalities.

Given that they extensively map the literature on underexplored topics, scoping reviews were the method that aligned with the purpose of this study^15^^,^^18^.

Prior to conducting the study, a research protocol detailing the methods employed was registered and is available at: https://doi.org/10.17605/OSF.IO/2UCSA.

Search strategy 

Evidence gathering was systematized in three steps: (i) initial search for scientific articles about SISVAN Web held on the PubMed and Latin American and Caribbean Health Sciences Literature (LILACS) databases to identify words present in titles, abstracts and indexing terms and to build the search strategy; (ii) search in all databases (PubMed, LILACS, Scopus, Web of Science, Cumulative Index to Nursing and Allied Health Literature - CINAHL, *Biblioteca Digital Brasileira de Teses e Dissertações* (BDBTD) and *Google Acadêmico*[Google Scholar]); and (iii) retrospective search for citations in the reference lists of literature reviews and grey literature^19^. 

The following search strategy was adopted, combining terms with AND and OR Boolean operators: ("sisvan" OR "food and nutrition* surveillance system" OR "nutrition* surveillance system" OR "food surveillance system") AND ("brazil" OR "brazilian"). The search strategy was used in all the databases except *Google Acadêmico* (Supplementary Table 1). The search was conducted on July 31, 2024, and updated on May 21, 2025. There were no restrictions on publication date or language.

Evidence selection 

Two independent researchers conducted the evidence selection in sequential steps defined in the protocol (steps 7 to 12), with support from the free Rayyan platform^20^. Primary studies (without design restriction) and literature reviews, published as scientific articles, undergraduate or residency final papers, master's dissertations or doctoral theses, that had undergone a peer review criterion and produced evidence from quantitative information based on SISVAN Web were included. Articles derived from undergraduate or residency final papers, dissertations and theses were used instead of the original source so as to avoid duplication.

We excluded technical documents and reports, opinion articles, editorials, letters, advocacy materials and policy briefs, as well as research that was not based on SISVAN Web data or that used SISVAN-*Indígena* data; research on SISVAN data quality or performance (e.g., coverage); intervention studies; and qualitative investigations (e.g., perception of health servise professionals or managers about SISVAN).

Data extraction and analysis 

Two researchers extracted data from ten primary studies to test the extraction spreadsheet. Only the principal researcher extracted the following information from the selected documents, using Google Sheets software (Alphabet Inc., California, United States): author(s), study name, year of publication, type of literature, journal, objectives, location, level of data disaggregation, data typology, design, population, SISVAN indicators, data source, and methods, in an interactive process with the team.

Regarding population classification, the same research could be included in several categories, depending on the stratification employed. For example, the same study may have evaluated multiple age groups or data source systems (*Bolsa Família* Program Management System, e-SUS PHC strategy, and SISVAN Web). Only population or data source stratifications applied to the description/analysis of nutrition status were considered; those used only for sample descriptions were not taken into account.

The publications were analyzed and categorized according to a glossary developed to standardize methodological elements (Supplementary Table 2)^21^^-^^24^.

## Results 

Figure 1 contains the flowchart^25^ with the sequential steps adopted to select the scientific literature that comprised this scoping review. In total, one systematic review was found, returning one eligible primary study, and two scientific articles were identified via dissertation/thesis.

The results presented here are based on the analysis of 161 publications that investigated the nutrition status component of SISVAN Web, either alone (n=123) or in addition to its food consumption component (n=38). Studies that assessed only the food consumption component were excluded (n=38).

Although the SISVAN Web platform was launched in 2007^4^, the first two publications date from 2011, increasing modestly until 2020. From 2021 onwards, a significant increase in publications was observed, especially in 2023. Scientific articles predominated, followed by course or residency final papers, while book chapters, dissertations and theses were less frequent (Figure 2).

Regarding the structure of the epidemiological data, 82.0% used aggregated secondary data from the consolidated SISVAN Web annual reports, with the exception of two studies that used monthly reports^26^^,^^27^. Of the total, 59.0% of the studies were classified as longitudinal, and 39.8% as cross-sectional; 64.0% were exploratory, and 36.0% analytical (Table 1).

**Figure 1:**
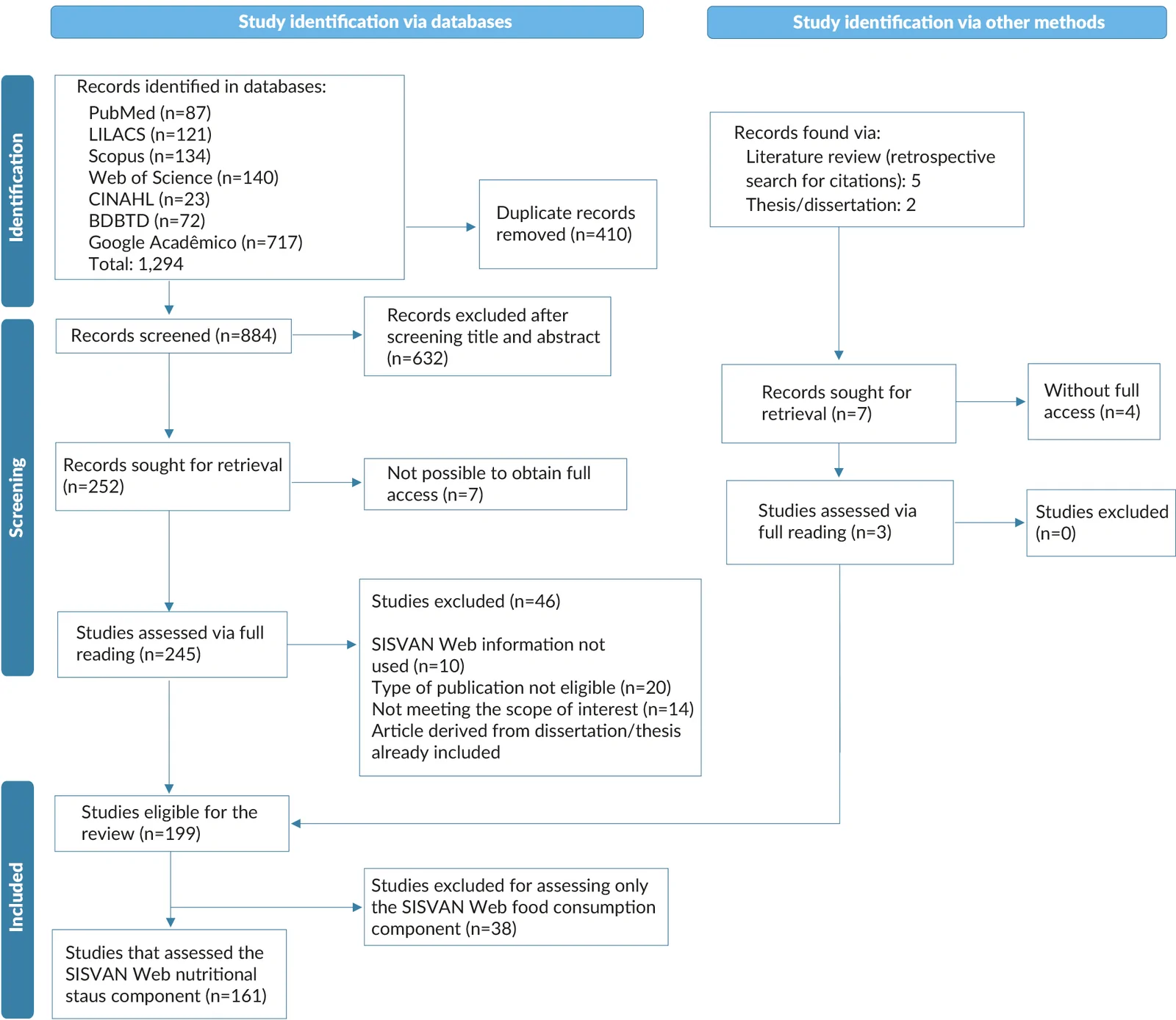
Selection process for studies eligible for inclusion in the scoping review, used in the two bibliographic searches

**Figura 2:**
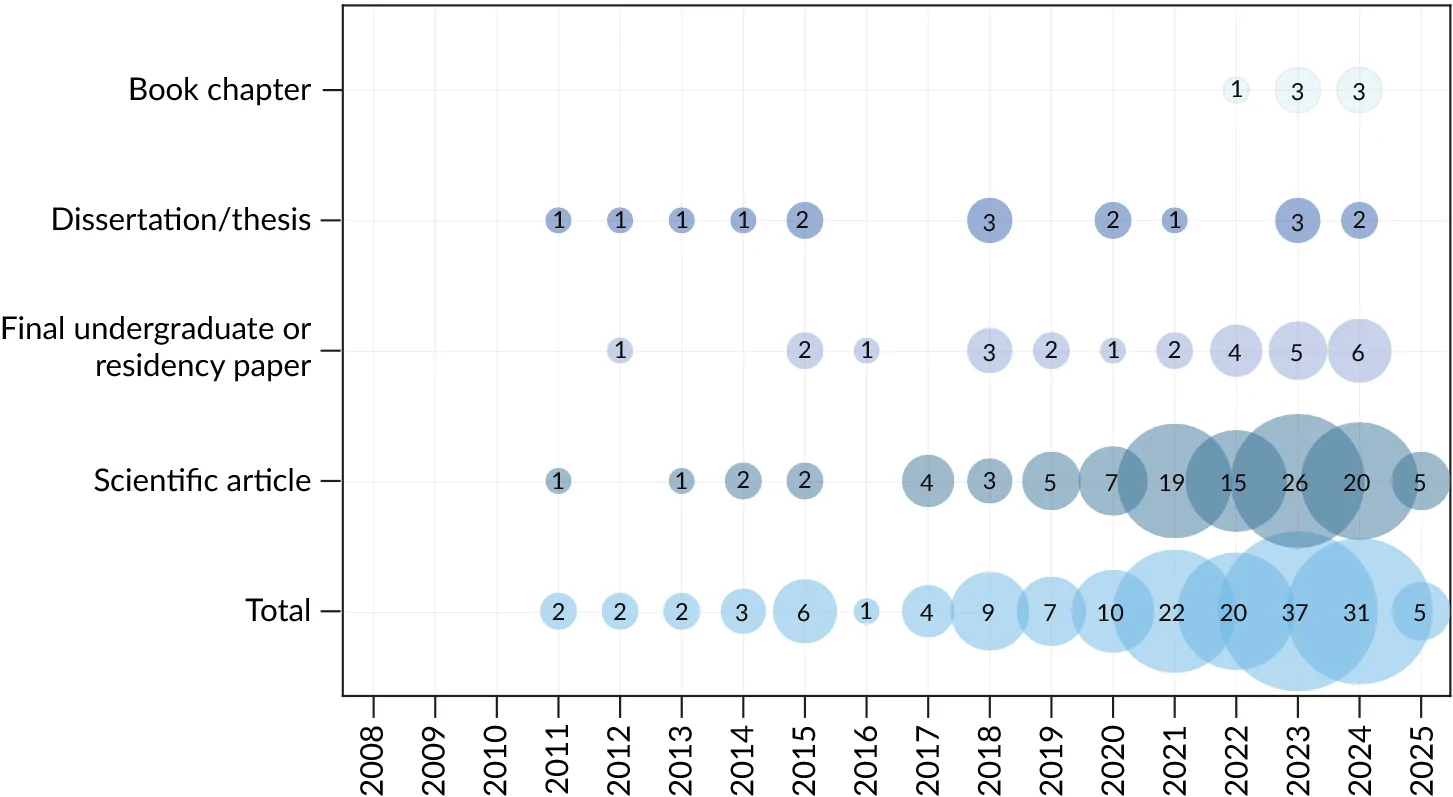
Number of publications per year by type of literature (n=161)** **

**Table 1:** Characterization of the methodology used in the publications analyzed (n=161)

Classification axis	n (%)
Unit of analysis	
Aggregate	132 (82.0)
Individualized	29 (18.0)
Temporality	
Longitudinal	95 (59.0)
Cross-sectional	64 (39.8)
Mixed^a^	2 (1.2)
Analysis type	
Descriptive or exploratory	104 (64,6)
Analytical	57 (35,4)
Unit of analysis: aggregate (n=132)	
Subtype (ecological) | temporality	
Time-series or longitudinal	86 (65.2)
Sectional or cross-sectional	44 (33.3)
Mixed^a^	2 (1.5)
Subtype (ecological) | unit of analysis	
Geographical area (municipalities, health regions, federative units or macro-regions)	126 (95.5)
Institutional (health establishment)	6 (4.5)
Subtype (ecological) | unit of analysis	
Multiple groups (two or more locations or institutions)	87 (65.9)
Single group (one location or institution)	45 (34.1)
Unit of analysis: individualized (n=29)	
Subtype (individual) | temporality	
Survey	16 (55.2)
Retrospective cohort	9 (31.0)
Repeated cross-sectional	4 (13.8)

a A thesis^77^ is a final course paper^140^ incorporating analyses that meet both designs (longitudinal and cross-sectional).

Among the studies with aggregated data (n=132), 95.5% used information from geographic databases, and 4.5% used reports from health establishments. The majority compared frequencies or trends between groups (locations or institutions) (65.9%) and employed an aggregated-observational-longitudinal design (65.2%). An increase in publications with an ecological design was observed, with 23 studies in 2023. Of the total (n=88), 46 were descriptive in nature. The remainder (n=42) were analytical; of these, 40 estimated trends in SISVAN indicators over time.

Among the 29 designs with individualized data, there were 16 cross-sectional^28^^-^^43^, nine historical cohort^44^^-^^52^ and four repeated cross-sectional studies^53^^-^^56^. Three used information from the School Health Program^32^^,^^40^^,^^43^; three from health establishments^38^^,^^39^^,^^44^; and one used microdata input to SISVAN Web by the nutritionist responsible for the municipal element of the National School Meals Program^28^.

Sixty-nine 69 studies used demographic information, mostly sex (n=61), followed by race/color (n=15), belonging to traditional peoples/communities (n=4), and schooling (n=3). Among the three studies with this latter aspect, one examined administrative data on children under 6 months according to daycare attendance^29^; another classified elderly people who attended or did not attend school^37^; and the last one selected only "primary education" from SISVAN Web for school data^57^. The four studies with traditional peoples/communities were published between 2021 and 2024, and three worked exclusively with information on these populations^30^^,^^54^^,^^58^^,^^59^ (Table 2).

**Table 2:** Populational characterization of the studies analyzed (n=161)

Stratification	n (%)
Demographic aspects (n=69; 42.9%)	
Sex^a^	61 (37.9)
Race/color	15 (9.3)
Traditional peoples and communities	4 (2.5)
Schooling	3 (1.9)
Data origin (n=40; 24.8%)	
*Bolsa Família* Program beneficiaries^b^	33 (20.5)
Food and Nutrition Surveillance System (SISVANn Web)	6 (3.7)
National Primary Health Care central information hub (e-SUS strategy)	2 (1.2)
Phase or event in life course (n=161)	
Infants (0-2 years)	12 (7.5)
(0-6 months)	9 (5.6)
(6-24 months)	10 (6.2)
Early childhood (0-5 years)	45 (28.0)
6 months-5 years	1 (0.6)
2-5 years	18 (11.2)
School children (5-10 years)	30 (18.6)
0-10 years	10 (6.2)
5-7 years	2 (1.2)
7-10 years	1 (0.6)
0-7 years	4 (2.5)
Adolescents (10-19 years)	28 (17.4)
Adults (20-59 years)	32 (19.9)
Elderly (60 years or over)	22 (13.7)
Prgnant women (any age)	18 (11.2)
Pregnant adolescents	8 (5.0)
Pregant adults	6 (3.7)

a Pregnant women were classified as "not applicable" to stratification by sex; ^b^The *Bolsa Família* Progam was replaced by the *Auxílio Brasil* Program in 2021, returning in June 2023.

Of the studies with race/color stratification, ten used all the SISVAN categories^29^^,^^37^^,^^40^^,^^50^^,^^54^^,^^60^^-^^64^. Two compared Black and non-Black populations^65^ or Black/mixed-race and White populations^66^; and three focused only on the non-village indigenous population, since monitoring of village populations is done through SISVAN-*Indígena*^67^^-^^69^. Seven studies reported missing race/color records in some of the data^37^^,^^50^^,^^61^^-^^63^^,^^70^^,^^71^.

Childhood stood out as the most studied life stage, particularly up to 5 years of age, followed by school-aged children, preschoolers and infants. A smaller number of analyses focused on more stratified age groups within childhood. The four studies with data from 0-7 years of age involved beneficiaries of the *Bolsa Família* Program. A smaller proportion studied adults, adolescents, the elderly and pregnant women.

Most studies that stratified the type of follow-up used the *Bolsa Família* Program Management System. Of these, 25 studied only beneficiaries, and eight compared the nutrition status of this group with that of all users. Studies that used data from SISVAN Web itself or the e-SUS PHC strategy did not compare nutrition indicators between systems nor justify the choice^57^^,^^68^^,^^72^^-^^74^, except for one thesis^56^ and one dissertation^75^ that analyzed data prior to the implementation of the latter system.

It was possible to identify the Federative Unit studied in 124 of the documents. Twelve studies covered all the Brazilian states. Among the others, there were one to 10 studies per state, except Bahia, Minas Gerais and Pernambuco, which stood out with 11 to 19 publications. A further 37 studies did not specify the Federative Unit, as they examined national data, macro-regions or groupings such as municipalities on the Brazilian border^76^ and Intermediate Regions of Urban Articulation^77^.

Most publications targeting children and adolescents assessed body mass index-for-age, followed by height-for-age. In the case of studies with adults, the elderly or pregnant women, the only index available via SISVAN Web was body mass index (Table 3).

Nine studies with individualized data explored other nutrition indicators, which is only possible with this type of unit of analysis. In general, the authors employed diverse designs (retrospective cohort, cross-sectional, and repeated cross-sectional) to analyze mean and percentile distributions of weight and height measurements; child growth trajectories (weight, height, and body mass index in unadjusted values and z-scores); catch-up growth (growth recovery according to z-scores); and gestational weight gain. Some studies related these indicators to sociodemographic variables, such as sex and maternal race/color, food consumption markers used in primary health care, and contextual aspects of municipalities^30^^,^^42^^,^^45^^-^^47^^,^^51^^-^^53^^,^^56^.

**Table 3:** Characterization of the nutrition status indicators used in the literature reviewed (n=161)

Nutrition indicator	n (%)
Children and adolescents	
Body mass index-for-age	96 (59.6)
Height-for-age	53 (32.9)
Weight-for-age	37 (23.0)
Weight-for-height	24 (14.9)
Adults, elderly and pregnant people	
Body mass index	51 (31.7)
Other^a^	9 (5.6)

a Studies using individualized data from the Food and Nutrition Surveillance System that analyzed nutrition status indicators not available in SISVAN Web public reports.

Regarding the statistical techniques used to estimate trends among studies with an aggregate-observational-longitudinal design, nine studies employed simple linear regression models^66^^,^^74^^,^^77^^-^^83^. One of them had a predictive character and estimated frequency of obesity in adults for 2025 and 2030, investigating the influence of the COVID-19 pandemic^83^.

Most publications employed the Prais-Winsten generalized linear regression technique (n=24), with the first two dating from 2021^84^^,^^85^. From that period onwards, use of this method increased^9^^,^^10^^,^^27^^,^^67^^,^^76^^,^^86^^-^^101^. The use of this approach was identified in one article with a repeated individualized-observational-cross-sectional design^54^. Furthermore, six studies used the Joinpoint Trend Analysis program, created by the National Cancer Institute^102^, to quantify trends^63^^,^^103^^-^^107^.

The interrupted time-series approach was found to be applied in an article that investigated the effect of the COVID-19 pandemic on malnutrition indicators in infants registered with the *Bolsa Família* Program, using the Prais-Winsten technique and an autoregressive, integrated and moving average model for seasonal time-series, using monthly SISVAN Web reports^27^.

Twelve publications were identified that aimed to study the impact of the COVID-19 health crisis^108^ on nutrition outcomes, with children being the most studied population. Of these, eight employed longitudinal ecological designs, six were descriptive^70^^,^^109^^-^^113^ and two were analytical^27^^,^^83^. The others had a descriptive cross-sectional character, one being a survey^33^ and three being cross-sectional ecological studies^64^^,^^114^^,^^115^.

The SISVAN coverage variable was reported in 29 of the 161 documents and was calculated in different ways. Among the studies that estimated temporal trends (n=41), 17 calculated SISVAN coverage. Of these, four used it to adjust regression models, based on the understanding that variations in coverage can explain part of the trend estimates of outcomes due to the relationship with case reports or health service access/quality^54^^,^^87^^,^^89^^,^^93^.

Some publications employed exploratory spatial data analyses on individualized^53^ or aggregated SISVAN information - thematic maps (n=12), global Moran's index and local Moran's index - using various geographic information and analysis systems at one^77^^,^^91^^,^^116^^-^^120^ or more points in time^89^^,^^98^^,^^105^^,^^121^. Three studies applied georeferencing/geocoding techniques to spatialize nutrition deviations at municipal scales, either through geographic coordinates of individual addresses^33^^,^^41^, or through aggregated SISVAN data, locating health establishments according to neighborhoods within municipalities^70^.

## Discussion 

This was the first scoping review on the use of the SISVAN Web system in investigating the nutrition profile of the population cared for by the Brazilian Unified Health System (SUS). Early childhood was the most studied phase, especially regarding body mass index-for-age. Most studies stratified the analyses by sex, while race/color and education level were less explored and presented limitations. *Bolsa Família* Program beneficiaries were the most studied group regarding the origin of the data. Research was concentrated in the states of Pernambuco, Minas Gerais and Bahia. Ecological designs predominated, with an increase in robust time-series studies. Some researchers used individualized SISVAN data, which expanded its analytical potential.

A limitation of this review was the absence of critical appraisal of the studies, potentially leading to selection bias, although this stage is not expected in scoping reviews. However, the volume of publications reviewed allowed us to meet the comprehensive exploratory purpose expected in this type of synthesis^15^^,^^16^^,^^18^. Analyzing the extensive volume of evidence presented a methodological challenge. Ideally, data extraction should be conducted by two or more researchers^15^; however, the collaborative processes adopted helped to minimize potential biases in data extraction and categorization.

The increasing applicability of SISVAN in scientific production may have been driven by national computerization in 2007, which brought operational improvements, favoring its consolidation and expansion of its coverage. By 2014, the system was recording information on all the Brazilian municipalities, although the largest volume of data was still concentrated on childhood^122^. Academic interest in this system also grew, evidenced by the increase in publications, especially after 2021. The increase in food insecurity, aggravated by the political and economic crises, has had repercussions on the living conditions of the population. It is known that this scenario affects and is reflected in the health status of individuals, putting them at risk of various forms of malnutrition^93^^,^^123^.

The increase in ecological research is consistent with the operational ease and low cost associated with secondary information, while use of anonymized individualized data requires its authorized provision by the Ministry of Health or access to the restricted module of the platform^7^, thus limiting its use by researchers. However, both types of data offer potential: individualized data are considered ideal for making inferences about associated or causal factors of health problems, while ecological data allow for understanding of phenomena at a more comprehensive level of determination, by capturing structural conditioning factors. When combined in hierarchical models, as observed in the reviewed works, they can provide relevant understanding of the phenomena of interest, this being a significant application of individualized SISVAN Web information^21^^,^^22^^,^^24^.

This system can signal social inequalities through its sociodemographic variables^70^^,^^124^, which are still underrepresented in aggregate studies, with the exception of sex. On the SISVAN data collection form, only sex and race/color are mandatory fields; belonging to traditional peoples and communities has been an available field since 2013^5^^,^^30^. Studies have shown shortcomings in the race/color field^37^^,^^50^^,^^61^^-^^63^^,^^70^^,^^71^, as well as lower SISVAN coverage for the Black population^65^ or possible classification or recording errors of this variable^54^.

However, since 2017, the national primary health care central information hub has been the preferred method for submitting food and nutrition surveillance data, instead of direct data entry to the SISVAN Web platform, which will be discontinued as the implementation of the e-SUS strategy progresses^12^^,^^125^^,^^126^. This interface is opportune for improving the quality of information held on SISVAN and strengthening use of sociodemographic information in gaining understanding of the social determinants of health^124^.

Regarding nutrition indicators, one study reported a preference for weight-for-age for children under 5 years old, as it eliminates the need to measure height, minimizing errors in estimates and encompassing a larger number of children with data held on SISVAN^103^. In the case of pregnant women, the Ministry of Health has adopted gestational weight gain as a diagnostic criterion, although SISVAN Web still uses body mass index per gestational week to consolidate data from this population^3^. The non-mandatory nature of filling in the pre-gestational weight field on SISVAN may result in a significant part of this data being missing^53^.

*Bolsa Família* Program data were the most used, consistent with the historical link between nutrition surveillance actions and government assistance programs, such as the *Bolsa Família* Program since 2004, and the linking of this system with SISVAN Web since it was launched^4^^,^^127^^,^^128^. In 2020, compliance with and recording of the *Bolsa Família* Program health conditionalities, including nutrition assessment, stopped being mandatory. Between 2021 and 2022, this program was replaced by the *Auxílio Brasil* Program, but was reinstated in June 2023. Understanding the impact of these changes on the SISVAN indicators is necessary^129^^-^^131^.

Studies using e-SUS strategy data are incipient, possibly due to the recent interface between the systems, begun in 2017^6^^,^^7^^,^^125^. This measure increased the number of food consumption records input via this system between 2015 and 2019. There is no evidence on nutrition indicators, and more in-depth evaluation of the effects of this strategy on SISVAN performance as a whole is necessary^12^.

Expansion of ecological time-series designs has been made possible by the volume of information regularly stored on SISVAN Web since its inception. There has been a growing use of robust statistical techniques to estimate temporal trends, such as the Prais-Winsten methodology, which is indicated for this purpose because it considers serial autocorrelation present in population measurements. This characteristic is contrary to assumptions required in traditional regression models, which limits their use in temporal analyses by potentially inducing incorrect interpretations of phenomena^132^^-^^134^. Centralizing the "year" variable to deal with autocorrelation is not recommended, as was identified in one of the documents^80^^,^^132^.

The use of the Prais-Winsten approach has grown in national studies involving various indicators of health, mortality and service performance^135^. The first application of this technique to SISVAN data dates from 2020, in an evaluation of the system's coverage for children in the Northern region of Brazil, between 2008 and 2017^8^ (search in LILACS and PubMed databases in August 2025 with the terms "prais-winsten" AND "sisvan"). This method was also applied to data from the Chronic Disease Risk and Protective Factors Surveillance Telephone Survey, another food and nutrition surveillance strategy^136^^-^^138^.

In addition to their use in epidemiological surveillance, temporal studies can reveal the effects of events or public policies. For this purpose, routinely collected data, such as SISVAN data, are appropriate as they enable long time-series to be built. Interrupted time-series analysis, considered the most robust quasi-experimental model for this purpose, although still incipient in the area of nutrition surveillance, can assess longitudinal effects of events/interventions on food and nutrition indicators^133^^,^^134^^,^^139^.

Research using spatial analysis and geocoding resources highlights important methodological tools for epidemiology. Freely accessible geographic information systems can facilitate their use with SISVAN data, contributing to spatial visualization of food and nutrition problems and knowledge of priority territories for interventions and surveillance measures.

This scoping review made it possible to identify elements to be explored in future research: (i) trend estimates with SISVAN Web data based on standardized and robust methods, for the various phases/events of the life course and geographic locations; (ii) longitudinal analyses that consider the effect of population events/interventions on nutrition indicators will increase the applicability of nutrition surveillance information; (iii) spatial analyses and geocoding on an intramunicipal scale enable local problems to be monitored as well as targeted effective efforts; (iv) better understanding of the effect of interoperability between SISVAN Web and the national PHC central information hub and the impact of changes in monitoring *Bolsa Família* Program conditionalities on system indicators; (v) systematizing missing demographic information in the macrodata, with the aim of informing sectors responsible for managing the platform; and (vi) incorporating hierarchical models into the microdata to investigate individual and contextual determinants of nutrition problems.

This review presented the state of the art in scientific production and reconnoitered the field of studies on nutrition surveillance via SISVAN, methodologically characterizing a broad body of evidence and identifying the use of information from this system. It is expected that the findings will contribute to informing future research and supporting the improvement of SISVAN.

## Data Availability

The data extracted from the selected documents have been stored in Google Sheets software (Alphabet Inc., California, United States) and will be available upon request to the corresponding author.
