# Supplementary material for: Use of the Food and Nutrition Surveillance System in epidemiological studies on the nutrition profile of the Brazilian population: a scoping review, Brazil, 2008-2025
Source: Epidemiol Serv Saude. 2026 Jul 26;35:e20251036. doi: 10.1590/S2237-96222026v35e20251036.en (PMC13686302; doi:10.1590/S2237-96222026v35e20251036.en)
Supplement: Supplementary file 1 [file 2237-9622-ress-35-e20251036-sup-en.pdf]

Supplementary Table 1. Search strategies adopted in the databases used in the scoping review

Database	Search strategy
PubMed	("sisvan" OR "food and nutrition* surveillance system" OR "nutrition* surveillance system" OR "food surveillance system") AND ("brazil" OR "brazilian")
Literatura Latino- Americana e do Caribe em Ciências da Saúde (Lilacs)	("sisvan" OR "food and nutrition* surveillance system" OR "nutrition* surveillance system" OR "food surveillance system") AND brazil*
Scopus	("sisvan" OR "food and nutrition* surveillance system" OR "nutrition* surveillance system" OR "food surveillance system") AND brazil*
Web of Science	("sisvan" OR "food and nutrition* surveillance system" OR "nutrition* surveillance system" OR "food surveillance system") AND brazil*
Cumulative Index to Nursing and Allied Health Literature – CINAHL	("sisvan" OR "food and nutrition* surveillance system" OR "nutrition* surveillance system" OR "food surveillance system") AND brazil*
Biblioteca Digital Brasileira de Teses e Dissertações	("sisvan" OR "food and nutrition* surveillance system" OR "nutrition* surveillance system" OR "food surveillance system") AND brazil*
Google Acadêmico	“sisvan web” (no citations)

Supplementary 2. Glossary of classification of the methodology used in the publications included in the scoping review

Unit of analysis	Position of the investigator	Design temporality		Design name	Ecological subtype	Ecological subtype
Aggregate (data express collectivities or geographic areas)	Observational[a]	Cross-sectional[c]		Ecological sectional	1. Territorial base/ geographic area (e.g., districts, municipalities, states, nations)<br>2. Institutional (e.g., collective organizations, schools, health centers and services)	1. Multiple groups<br>2. Single group
		Longitudinal[d]		Ecologic time-series		
	Intervention[b]	Longitudinal[d]		Community trial	-	-
Individualized (data express attributes of the individual singly)	Observational[a]	Cross-sectional[c]		Survey[c]	-	-
				Repeated cross-sectional[c]		
		Longitudinal[d]	Prospective	Cohort[e]	-	-
			Retrospective	Time-series cohort[f]	-	-
				Case-control	-	-
	Intervention[b]	Longitudinal[d]		Clinical trial	-	-

**Analysis type[g]**	
Descriptive or exploratory	Their purpose is to formulate hypotheses. They describe and compare the frequency distribution of outcomes of interest in populations at a given point in time or over time. They encompass association/correlation studies and studies that describe trends in indicators over time, without the intention of testing hypotheses.
Analytical	Its purpose is to test hypotheses. It tests associations between exposure factors and outcomes of interest to investigate determinants of health conditions/problems in populations. It encompasses studies that estimate trends in indicators over time or cross-sectional designs that estimate prevalence ratios.

Notes: [a]The investigator does not interfere in the specific objects studied or in the processes of producing outcomes in the populations; [b]The investigator interferes in the study processes in a systematic and controlled manner (experimentation); [c]Data collection or acquisition is performed simultaneously, so that the factor and outcome studied are observed at the same moment in time. It also aims to describe and compare rates of health indicators between populations at a point in time (surveys) or over a period (repeated cross-sectional studies). In the latter category, data are obtained from different individuals, but from the same population of interest, at various times, thus building a time series, which makes this design similar to longitudinal studies; [d]Data collection or acquisition occurs on a serial/temporal scale, so that the factor and outcome studied are observed at successive points in time. It also aims to compare, in defined populations, rates of health indicators over time; [e]The investigation takes place in parallel with the phenomenon being studied, so that the start of the research coincides in time with the start of the cohort; [f]The investigation takes place after the evolution of the outcomes in the cohort studied, so that the cohort is retrospective; [g]The division between analysis types may not be conceptually rigid; in reality, a spectrum exists between descriptive and analytical subtypes.
