# Supplementary material for: Use of the Food and Nutrition Surveillance System in epidemiological studies on the nutrition profile of the Brazilian population: a scoping review, Brazil, 2008-2025
Source: Epidemiol Serv Saude. 2026 Jul 26;35:e20251036. doi: 10.1590/S2237-96222026v35e20251036.en (PMC13686302; doi:10.1590/S2237-96222026v35e20251036.en)
Supplement: Supplementary file 2 [file 2237-9622-ress-35-e20251036-sup-pt.pdf]

Tabela Suplementar 1. Estratégias de busca adotadas nas bases de dados utilizadas na revisão de escopo

**Base de dados**	**Estratégia de busca**
PubMed	("sisvan" OR "food and nutrition* surveillance system" OR "nutrition* surveillance system" OR "food surveillance system") AND ("brazil" OR "brazilian")
Literatura Latino- Americana e do Caribe em Ciências da Saúde (Lilacs)	("sisvan" OR "food and nutrition* surveillance system" OR "nutrition* surveillance system" OR "food surveillance system") AND brazil*
Scopus	("sisvan" OR "food and nutrition* surveillance system" OR "nutrition* surveillance system" OR "food surveillance system") AND brazil*
Web of Science	("sisvan" OR "food and nutrition* surveillance system" OR "nutrition* surveillance system" OR "food surveillance system") AND brazil*
Cumulative Index to Nursing and Allied Health Literature – CINAHL	("sisvan" OR "food and nutrition* surveillance system" OR "nutrition* surveillance system" OR "food surveillance system") AND brazil*
Biblioteca Digital Brasileira de Teses e Dissertações	("sisvan" OR "food and nutrition* surveillance system" OR "nutrition* surveillance system" OR "food surveillance system") AND brazil*
Google Acadêmico	“sisvan web” (sem citações)

Tabela Suplementar 2. Glossário de classificação metodológica das publicações incluídas na revisão de escopo

**Unidade analítica**	**Posição do investigador**	**Temporalidade do delineamento**		**Denominação do delineamento**	**Subtipo ecológico**	**Subtipo ecológico**
Agregado (dados expressam coletividades ou áreas geográficas)	Observacional[a]	Transversal[c]		Ecológico seccional	1. Base territorial/área geográfica (exemplo: distritos, municípios, estados, nações)<br><br>2. Institucional (exemplo: organizações coletivas, escolas, unidades e serviços de saúde)	1. Múltiplos grupos<br><br>2. Grupo único
		Longitudinal[d]		Ecológico de série temporal		
	Intervenção[b]	Longitudinal[d]		Ensaio comunitário	-	-
Individualizado (dados expressam atributos do indivíduo singularmente)	Observacional[a]	Transversal[c]		Inquérito[c]	-	-
				Transversal repetido[c]		
		Longitudinal[d]	Prospectivo	Coorte[e]	-	-
			Retrospectivo	Coorte histórica[f]	-	-
				Caso-controle	-	-
	Intervenção[b]	Longitudinal[d]		Ensaio clínico	-	-

**Tipologia analítica[g]**	
Descritivo ou exploratório	Tem por objetivo formular hipóteses. Descreve e compara a distribuição das frequências de desfechos de interesse nas populações em um dado momento ou através do tempo. Abrange estudos de associação/correlação e estudos que descrevem tendências de indicadores ao longo do tempo, sem a pretensão de testar hipóteses.
Analítico	Tem por objetivo testar hipóteses. Testa associações entre fatores de exposição e desfechos de interesse para investigar determinantes das condições/agravos de saúde em populações. Abrange estudos que estimam tendências de indicadores ao longo do tempo ou delineamentos transversais que estimam razões de prevalência.

Nota: [a]Não há interferência do investigador nos objetos concretos estudados e nos processos de produção de desfechos nas populações; [b]O investigador interfere nos processos do estudo, de maneira sistemática e controlada (experimentação); [c]A coleta ou a obtenção do dado é realizada ao mesmo tempo, de modo que o fator e o desfecho estudados são observados no mesmo momento histórico. Também objetiva descrever e comparar taxas de indicadores de saúde entre populações em um momento do tempo (inquéritos) ou durante um período (transversal repetido). Nessa última categoria, são obtidos dados de indivíduos diferentes, mas vindos da mesma população de interesse, em vários momentos, construindo uma série temporal, o que assemelha esse desenho aos de temporalidade longitudinal; [d]A coleta ou a obtenção do dado ocorre em uma escala serial/temporal, de modo que o fator e o desfecho estudados são observados em momentos históricos sucessivos. Também objetiva comparar, em populações definidas, taxas de indicadores de saúde através do tempo; [e]A investigação ocorre paralelamente ao fenômeno estudado, de modo que o início da pesquisa coincide historicamente com o início da coorte; [f]A investigação ocorre após a evolução dos desfechos na coorte estudada, de modo que há uma reconstrução histórica da coorte; [g]A divisão entre tipologias de análise pode não ser conceitualmente rígida, existindo na realidade um espectro entre os subtipos descritivo versus analítico.
