# Supplementary material for: Use of the Food and Nutrition Surveillance System in epidemiological studies on the nutrition profile of the Brazilian population: a scoping review, Brazil, 2008-2025
Source: Epidemiol Serv Saude. 2026 Jul 26;35:e20251036. doi: 10.1590/S2237-96222026v35e20251036.en (PMC13686302; doi:10.1590/S2237-96222026v35e20251036.en)
Supplement: Translation

# Translation: O uso do Sistema de Vigilância Alimentar e Nutricional em estudos epidemiológicos sobre o perfil nutricional da população brasileira: revisão de escopo, Brasil, 2008-2025

**DOI:** 10.1590/S2237-96222026v35e20251036.pt

**Authors:** Caroline de Oliveira Gallo, Luiza Navarro de Azevedo, Patricia Constante Jaime

**Affiliations:** 1 Universidade de São Paulo, Departamento de Nutrição, São Paulo, SP, Brasil Universidade de São Paulo Departamento de Nutrição São Paulo SP Brasil; 2 Universidade de São Paulo, Núcleo de Pesquisas Epidemiológicas em Nutrição e Saúde, São Paulo, SP, Brasil Universidade de São Paulo Núcleo de Pesquisas Epidemiológicas em Nutrição e Saúde São Paulo SP Brasil

**Keywords:** Vigilância Alimentar e Nutricional, Sistemas de Informação em Saúde, Estado Nutricional, Epidemiologia Nutricional, Revisão de Escopo

## Abstract

**Objetivos:**

Identificar e caracterizar a literatura científica sobre o uso do Sistema de Vigilância Alimentar e Nutricional (Sisvan) na descrição e na análise do perfil nutricional da população brasileira usuária do Sistema Único de Saúde.

**Métodos:**

Trata-se de revisão de escopo. A seleção dos documentos ocorreu de forma independente em etapas previamente definidas em protocolo de pesquisa. Incluíram-se estudos primários, revisões e literatura cinzenta que passaram por avaliação por pares e produziram evidências a partir de informações quantitativas sobre estado nutricional do Sisvan Web, de todas as populações e localizações. A extração dos dados ocorreu de forma padronizada, e as publicações foram analisadas segundo glossário desenvolvido para padronizar aspectos metodológicos.

**Resultados:**

Identificaram-se 1.294 registros e analisaram-se 161 após a aplicação dos critérios de elegibilidade. Houve incremento expressivo de publicações, especialmente a partir de 2021, destacando-se delineamentos ecológicos de séries temporais, que têm adotado métodos robustos para analisar dados do Sisvan. Identificaram-se 29 pesquisas que trabalharam dados individualizados, com recorte transversal ou coorte histórica. A fase da vida mais estudada foi a infância, particularmente até os 5 anos. Em 40 pesquisas, a origem do dado foi estratificada, e a maioria utilizou os provenientes do Programa Bolsa Família. Pernambuco, Minas Gerais e Bahia foram os estados mais estudados. Algumas pesquisas investigaram o efeito da pandemia de covid-19 em indicadores do Sisvan ou utilizaram métodos de georreferenciamento/análise espacial.

**Conclusão:**

Esta revisão apresentou o estado da arte da produção científica com uso de informações nutricionais do Sisvan e caracterizou aspectos metodológicos, informando pesquisas futuras.

## Introdução

A Política Nacional de Alimentação e Nutrição reconhece a vigilância alimentar e nutricional como qualificadora do cuidado no Sistema Único de Saúde (SUS)^1^. Para isso, o Sistema de Vigilância Alimentar e Nutricional (Sisvan) promove o diagnóstico alimentar e nutricional da população e identifica áreas geográficas e grupos sob risco, subsidiando a atenção no SUS e as decisões em saúde pública^2^. 

A prática da vigilância alimentar e nutricional ocorre principalmente na Atenção Primária à Saúde (APS). Baseada na avaliação antropométrica e do consumo alimentar e na triagem da Segurança Alimentar e Nutricional, tal prática é operacionalizada pelo sistema de informação em saúde Sisvan Web, criado em 2007 para centralizar a gestão das informações^3^^-^^5^. Essa plataforma recebe dados do centralizador nacional de informações da APS (estratégia e-SUS APS)^6^, do Sistema de Gestão do Programa Bolsa Família, além daqueles digitados diretamente na plataforma por gestores do SUS. Os dados são acessados em relatórios públicos, os quais contêm frequências das classificações nutricionais em áreas geográficas ou estabelecimentos de saúde na escala intramunicipal^5^, ou via módulo restrito por gestores e técnicos das Secretarias de Saúde, que acessam dados individualizados^7^.

A vigilância alimentar e nutricional tem sido objeto de interesse nas investigações científicas no campo da nutrição em saúde pública. Pesquisadores têm utilizado dados do Sisvan para fundamentar investigações sobre o perfil alimentar e nutricional da população assistida pelo SUS, o desempenho desse sistema e a qualidade dos dados coletados^8^^-^^14^. 

Apesar da importância desse sistema para gerar evidências no campo da vigilância e do potencial analítico dos dados coletados continuamente, não há estudos que sistematizem a literatura produzida. Torna-se relevante, portanto, mapear de forma sistematizada a produção científica baseada no Sisvan, para compreender a extensão e o tipo de evidência que vêm sendo produzidos e identificar lacunas, fortalezas e aspectos centrais no conhecimento sobre esse sistema de informação.

Os objetivos deste trabalho foram identificar e caracterizar a literatura científica sobre o uso do Sisvan Web na descrição e na análise do perfil nutricional da população brasileira. Busca-se compreender como as informações provenientes do Sisvan Web têm sido utilizadas na produção científica, identificando as populações mais estudadas no que concerne à faixa etária, à localização, ao sexo, à raça/cor e à escolaridade; os indicadores de estado nutricional mais avaliados; e os delineamentos e as análises de dados mais empregados nas pesquisas.

## Métodos 

Delineamento

Trata-se de revisão de escopo orientada pelo manual do Instituto Joanna Briggs e pela lista de verificação do Preferred Reporting Items for Systematic reviews and Meta-Analyses extension for Scoping Reviews^15^^-^^17^, bem como pelos elementos população-conceito-contexto.

População: pessoas atendidas na APS do SUS com dados consolidados no Sisvan Web, de todas as fases/evento do curso de vida, sexos, raças/cores e níveis de escolaridade.

Conceito: estudos epidemiológicos sobre o perfil alimentar e nutricional da população com dados consolidados no Sisvan Web.

Contexto: APS do SUS, cobrindo todo o território brasileiro, estados e municípios.

Por mapearem extensamente a literatura sobre temas pouco explorados, as revisões de escopo alinharam-se ao propósito deste trabalho^15^^,^^18^.

Previamente, registrou-se protocolo de pesquisa que detalha os métodos empregados, disponível em: https://doi.org/10.17605/OSF.IO/2UCSA.

Estratégia de busca 

A captação de evidências foi sistematizada em três passos: (i) busca inicial de artigos científicos sobre o Sisvan Web nas bases PubMed e Literatura Latino-Americana e do Caribe em Ciências da Saúde (Lilacs) para identificar palavras presentes em títulos, resumos e termos de indexação e construir a estratégia de busca; (ii) busca em todas as bases de dados (PubMed, Lilacs, Scopus, Web of Science, Cumulative Index to Nursing and Allied Health Literature - CINAHL, Biblioteca Digital Brasileira de Teses e Dissertações e Google Acadêmico); e (iii) busca retrospectiva por citações nas listas de referência de revisões da literatura e de literatura cinzenta^19^. 

Adotou-se a seguinte estratégia de busca combinando termos com operadores booleanos AND e OR: ("sisvan" OR "food and nutrition* surveillance system" OR "nutrition* surveillance system" OR "food surveillance system") AND ("brazil" OR "brazilian"), utilizada nas bases, exceto na Google Acadêmico (Tabela Suplementar 1). A busca ocorreu em 31 de julho de 2024 e foi atualizada em 21 de maio de 2025. Não houve limitações de data ou idioma de publicação.

Seleção das evidências 

Duas pesquisadoras independentes conduziram a seleção de evidências em etapas sequenciais definidas no protocolo (etapas 7 a 12), com suporte da plataforma livre Rayyan^20^. Incluíram-se estudos primários (sem restrição de delineamento) e revisões da literatura, publicados na forma de artigos científicos, trabalhos de conclusão de curso ou de residência, dissertações de mestrado ou teses de doutorado, que passaram por algum critério de avaliação por pares e produziram evidências a partir de informações quantitativas do Sisvan Web. Artigos derivados de trabalhos de conclusão de curso ou de residência, dissertações e teses substituíram a fonte original, o que evitou duplicidades.

Excluíram-se documentos e relatórios técnicos, artigos de opinião, editoriais, cartas, materiais de advocacy e *policy briefs*, assim como pesquisas que não trabalharam dados do Sisvan Web ou que utilizaram o Sisvan-Indígena; pesquisas sobre qualidade dos dados ou desempenho do Sisvan (por exemplo, cobertura); estudos de intervenção; e investigações qualitativas (por exemplo, percepção de profissionais ou gestores sobre o Sisvan). 

Extração e análise dos dados

Duas pesquisadoras extraíram dados de dez estudos primários para testar a planilha de extração. Somente a pesquisadora principal extraiu as seguintes informações dos documentos selecionados, utilizando o software Google Sheets (Alphabet Inc., Califórnia, Estados Unidos): autor(es), nome do estudo, ano de publicação, tipo de literatura, revista, objetivos, local, nível de desagregação e tipologia dos dados, delineamento, população, indicadores do Sisvan, origem do dado e métodos, em um processo interativo com a equipe.

Acerca da classificação populacional, uma mesma pesquisa pôde ser incluída em diversas categorias, dependendo da estratificação empregada. Exemplificando, um mesmo estudo pode ter avaliado múltiplas faixas etárias ou sistemas de origem do dado (Sistema de Gestão do Programa Bolsa Família, estratégia e-SUS APS e Sisvan Web). Consideraram-se somente estratificações populacionais ou de origem do dado aplicadas à descrição/análise do estado nutricional; as utilizadas apenas para descrições amostrais não foram contabilizadas. 

As publicações foram analisadas e categorizadas segundo glossário desenvolvido para padronizar elementos metodológicos (Tabela Suplementar 2)^21^^-^^24^.

## Resultados 

A Figura 1 apresentou o fluxograma^25^ com as etapas sequenciais adotadas para selecionar a literatura científica que compôs esta revisão de escopo. Ao todo, uma revisão sistemática foi encontrada, retornando um estudo primário elegível, e dois artigos científicos foram identificados via dissertação/tese.

Os resultados aqui apresentados basearam-se na análise de 161 publicações que investigaram o componente de estado nutricional do Sisvan Web isoladamente (n=123) ou adicionalmente ao componente de consumo alimentar (n=38). Pesquisas que avaliaram somente o componente de consumo alimentar foram excluídas (n=38). 

Embora a plataforma tenha sido lançada em 2007^4^, as primeiras duas publicações datam de 2011, aumentando modestamente até 2020. A partir de 2021, observou-se aumento expressivo de publicações, especialmente em 2023. Predominaram artigos científicos, seguidos pelos trabalhos de conclusão de curso ou de residência, enquanto capítulos de livro, dissertações e teses foram menos frequentes (Figura 2). 

Acerca da estrutura do dado epidemiológico, 82,0% utilizaram dados secundários agregados dos relatórios anuais consolidados do Sisvan Web, à exceção de dois trabalhos que utilizaram relatórios mensais^26^^,^^27^. Do total, 59,0% das pesquisas foram classificadas como longitudinais, e 39,8% como seccionais; "65% foram exploratórias, e 35% analíticas analíticas (Tabela 1). 

**Figura 1:** Processo de seleção dos estudos elegíveis para integrar a revisão de escopo, referente às duas buscas bibliográficas

**Figura 2:** Número de publicações por ano segundo tipo de literatura (n=161)** **

**Tabela 1:** Caracterização metodológica das publicações analisadas (n=161)

Eixo classificatório	n (%)
Unidade analítica	
Agregado	132 (82,0)
Individualizado	29 (18,0)
Temporalidade	
Longitudinais	95 (59,0)
Transversais	64 (39,8)
Misto^a^	2 (1,2)
Tipologia analítica	
Descritivo ou exploratório	104 (64,6)
Analítico	57 (35,4)
Unidade analítica: agregado (n=132)	
Subtipo (ecológico) | temporalidade	
Série temporal ou longitudinal	86 (65,2)
Seccional ou transversal	44 (33,3)
Misto^a^	2 (1,5)
Subtipo (ecológico) | unidade de análise	
Área geográfica (municípios, regiões de saúde, unidades federativas ou macrorregiões)	126 (95,5)
Institucional (estabelecimento de saúde)	6 (4,5)
Subtipo (ecológico) | unidade de análise	
Múltiplos grupos (duas ou mais localidades ou instituições)	87 (65,9)
Grupo único (uma localidade ou instituição)	45 (34,1)
Unidade analítica: individualizado (n=29)	
Subtipo (individualizado) | temporalidade	
Inquérito	16 (55,2)
Coorte histórica	9 (31,0)
Transversal repetido	4 (13,8)

a Uma tese^77^ e um trabalho de conclusão de curso^140^ incorporaram análises que atendem a ambos os delineamentos (longitudinal e transversal).

Entre os estudos com dados agregados (n=132), 95,5% usaram informações de bases geográficas, e 4,5% os relatórios de estabelecimentos de saúde. A maioria comparou frequências ou tendências entre grupos (localidades ou instituições) (65,9%) e empregou delineamento agregado-observacional-longitudinal (65,2%). Observou-se aumento de publicações com delineamento ecológico, com 23 pesquisas em 2023. Do total (n=88), 46 tiveram caráter descritivo. O restante (n=42) teve caráter analítico; destes, 40 estimaram tendências dos indicadores do Sisvan ao longo do tempo. 

Entre os 29 desenhos com dados individualizados, havia 16 transversais^28^^-^^43^, nove coortes históricas^44^^-^^52^ e quatro transversais repetidos^53^^-^^56^. Três usaram informações do Programa Saúde na Escola^32^^,^^40^^,^^43^; três de estabelecimentos de saúde^38^^,^^39^^,^^44^; e um utilizou microdados inseridos no Sisvan Web pela nutricionista responsável pelo Programa Nacional de Alimentação Escolar municipal^28^.

Ao total, 69 estudos utilizaram informações demográficas, a maioria sexo (n=61), seguido por raça/cor (n=15), pertencimento a povos/comunidades tradicionais (n=4) e escolaridade (n=3). Entre os três estudos com esse último aspecto, um trabalhou dados administrativos de menores de 6 meses segundo frequência em creche^29^; outro classificou idosos que frequentaram ou não a escola^37^; e o último selecionou somente "ensino fundamental" no Sisvan Web para dados de escolares^57^. Os quatro trabalhos com povos/comunidades tradicionais foram publicados entre 2021 e 2024, e três trabalharam exclusivamente informações dessas populações^30^^,^^54^^,^^58^^,^^59^ (Tabela 2). 

**Tabela 2:** Caracterização populacional das pesquisas analisadas (n=161)

Estratificação	n (%)
Aspectos demográficos (n=69; 42,9%)	
Sexo^a^	61 (37,9)
Raça/cor	15 (9,3)
Povos e comunidades tradicionais	4 (2,5)
Escolaridade	3 (1,9)
Origem do dado (n=40; 24,8%)	
Beneficiários do Programa Bolsa Família^b^	33 (20,5)
Sistema de Vigilância Alimentar e Nutricional (Sisvan Web)	6 (3,7)
Centralizador nacional de informações da Atenção Primária à Saúde (Estratégia e-SUS)	2 (1,2)
Fase ou evento do curso de vida (n=161)	
Lactentes (0-2 anos)	12 (7,5)
(0-6 meses)	9 (5,6)
(6-24 meses)	10 (6,2)
Primeira infância (0-5 anos)	45 (28,0)
6 meses-5 anos	1 (0,6)
2-5 anos	18 (11,2)
Escolares (5-10 anos)	30 (18,6)
0-10 anos	10 (6,2)
5-7 anos	2 (1,2)
7-10 anos	1 (0,6)
0-7 anos	4 (2,5)
Adolescentes (10-19 anos)	28 (17,4)
Adultos (20-59 anos)	32 (19,9)
Idosos (60 anos ou mais)	22 (13,7)
Gestantes (qualquer idade)	18 (11,2)
Gestantes adolescentes	8 (5,0)
Gestantes adultas	6 (3,7)

a Classificaram-se gestantes em "não se aplica" à estratificação por sexo; ^b^O Programa Bolsa Família foi substituído pelo Programa Auxílio Brasil em 2021, sendo novamente instituído em junho de 2023.

Dos trabalhos com estratificação de raça/cor, dez utilizaram todas as categorias do Sisvan^29^^,^^37^^,^^40^^,^^50^^,^^54^^,^^60^^-^^64^. Dois compararam populações negras e não negras^65^ ou pretas/pardas e brancas^66^; e três voltaram-se apenas à população indígena não aldeada, já que o monitoramento das aldeadas ocorre pelo Sisvan-Indígena^67^^-^^69^. Sete estudos relataram ausência de registro de raça/cor em parte dos dados^37^^,^^50^^,^^61^^-^^63^^,^^70^^,^^71^.

A infância destacou-se como a fase da vida mais estudada, particularmente até 5 anos, seguida dos escolares, pré-escolares e lactentes. Em menor número, houve análises específicas para faixas etárias mais estratificadas da infância. Os quatro estudos com dados de 0-7 anos foram com beneficiários do Programa Bolsa Família. Menor proporção estudou adultos, adolescentes, idosos e gestantes. 

A maioria das pesquisas que estratificou o tipo de acompanhamento utilizou o Sistema de Gestão do Programa Bolsa Família. Destas, 25 estudaram apenas beneficiários, e oito compararam o estado nutricional desse grupo com o de todos os usuários. Os trabalhos que utilizaram dados do próprio Sisvan Web ou da estratégia e-SUS APS não compararam indicadores nutricionais entre sistemas nem justificaram a escolha^57^^,^^68^^,^^72^^-^^74^, exceto uma tese^56^ e uma dissertação^75^ que analisaram dados anteriores à implementação desse último sistema. 

Em 124 documentos, foi possível identificar a Unidade Federativa estudada. Doze pesquisas abrangeram todos os estados. Entre as demais, houve de um a 10 estudos por estado, exceto Bahia, Minas Gerais e Pernambuco, que se destacaram com 11 a 19 publicações. Outros 37 estudos não especificaram a Unidade Federativa, por trabalharem dados nacionais, de macrorregiões ou de agrupamentos como municípios de fronteiras do país^76^ e Regiões Intermediárias de Articulação Urbana^77^.

A maioria das publicações com o público infantojuvenil avaliou o índice de massa corporal-para-idade, seguido da estatura-para-idade. Para estudos com adultos, idosos ou gestantes, o único índice disponibilizado pelo Sisvan Web foi o índice de massa corporal (Tabela 3). 

Nove pesquisas com informações individualizadas exploraram outros indicadores nutricionais, o que é possível apenas com esse tipo de unidade analítica. Em geral, os autores empregaram delineamentos diversos (coorte histórica, transversal e transversal repetido) para analisar distribuições médias e percentis de medidas de peso e altura; trajetórias de crescimento infantil (peso, estatura e índice de massa corporal em valores brutos e escore-z); *catch-up growth* (recuperação do crescimento segundo escore-z); e ganho de peso gestacional. Algumas pesquisas relacionaram esses indicadores com variáveis sociodemográficas, como sexo e raça/cor materna, marcadores de consumo alimentar utilizados na APS e aspectos contextuais dos municípios ^30^^,^^42^^,^^45^^-^^47^^,^^51^^-^^53^^,^^56^.

**Tabela 3:** Caracterização dos indicadores de estado nutricional empregados na literatura revisada (n=161)

Indicador nutricional	n (%)
Crianças e adolescentes	
Índice de massa corporal-para-idade	96 (59,6)
Estatura-para-idade	53 (32,9)
Peso-para-idade	37 (23,0)
Peso-para-estatura	24 (14,9)
Adultos, idosos e gestantes	
Índice de massa corporal	51 (31,7)
Outros^a^	9 (5,6)

a Estudos com dados individualizados do Sistema de Vigilância Alimentar e Nutricional que analisaram indicadores de estado nutricional não disponibilizados nos relatórios públicos do Sisvan Web.

Acerca das técnicas estatísticas utilizadas para estimar tendências entre os estudos com desenho agregado-observacional-longitudinal, nove pesquisas empregaram modelos de regressão linear simples^66^^,^^74^^,^^77^^-^^83^. Uma delas teve caráter preditivo e estimou a frequência de obesidade em adultos para 2025 e 2030, investigando a influência da pandemia de covid-19^83^.

A maioria das publicações empregou a técnica de regressão linear generalizada de Prais-Winsten (n=24), com as primeiras duas datadas de 2021^84^^,^^85^. A partir desse período, cresceu o uso desse método^9^^,^^10^^,^^27^^,^^67^^,^^76^^,^^86^^-^^101^. Identificou-se o uso dessa abordagem em um artigo com desenho individualizado-observacional-transversal repetido^54^. Ainda, seis pesquisas utilizaram o programa Joinpoint Trend Analysis, criado pelo National Cancer Institute^102^, para quantificar tendências^63^^,^^103^^-^^107^.

Encontrou-se aplicação da abordagem de séries temporais interrompidas em um artigo que investigou o efeito da pandemia de covid-19 sobre indicadores de má nutrição em lactentes inscritos no Programa Bolsa Família, utilizando a técnica de Prais-Winsten e um modelo autorregressivo, integrado e de médias móveis para séries com sazonalidade, por meio de relatórios mensais do Sisvan Web^27^.

Identificaram-se 12 publicações que objetivaram estudar o impacto da crise sanitária de covid-19^108^ sobre desfechos nutricionais, sendo o público infantil o mais estudado. Destas, oito empregaram desenhos ecológicos longitudinais, sendo seis descritivos^70^^,^^109^^-^^113^ e dois analíticos^27^^,^^83^. Os demais tiveram caráter transversal descritivo, sendo um inquérito^33^ e três ecológicos seccionais^64^^,^^114^^,^^115^.

A variável de cobertura do Sisvan foi relatada em 29 dos 161 documentos e calculada de formas distintas. Entre as pesquisas que estimaram tendências temporais (n=41), 17 calcularam a cobertura do Sisvan. Destas, quatro a utilizaram para ajustar os modelos de regressão, no entendimento de que variações na cobertura podem explicar parte das estimativas de tendência dos desfechos pela relação com notificações de casos ou acesso/qualidade dos serviços de saúde^54^^,^^87^^,^^89^^,^^93^.

Algumas publicações empregaram análises exploratórias de dados espaciais às informações individualizadas^53^ ou agregadas do Sisvan - mapas temáticos (n=12), índice de Moran global e índice de Moran local - utilizando diversos sistemas de informação e análise geográfica em um^77^^,^^91^^,^^116^^-^^120^ ou mais pontos no tempo^89^^,^^98^^,^^105^^,^^121^. Três trabalhos aplicaram técnicas de georreferenciamento/geocodificação para espacializar desvios nutricionais em escalas municipais, seja por meio de coordenadas geográficas de endereços individuais^33^^,^^41^, seja por meio de dados agregados do Sisvan, localizando estabelecimentos de saúde segundo bairros do município^70^.

## Discussão 

Tratou-se da primeira revisão de escopo sobre o uso do Sisvan Web na investigação do perfil nutricional da população assistida pelo SUS. A primeira infância foi a fase mais estudada, especialmente quanto ao índice de massa corporal-para-idade. A maioria dos trabalhos estratificou as análises por sexo, enquanto raça/cor e escolaridade foram menos explorados e apresentaram limitações. Beneficiários do Programa Bolsa Família constituíram o grupo mais estudado quanto à origem do dado. Concentraram-se pesquisas em Pernambuco, Minas Gerais e Bahia. Predominaram delineamentos ecológicos, com incremento de séries temporais robustas. Alguns pesquisadores utilizaram dados individualizados do Sisvan, o que ampliou seu potencial analítico.

Uma limitação deste trabalho recaiu na ausência de avaliação crítica dos estudos, incorrendo em possível seleção com viés, embora essa etapa não seja prevista em revisões de escopo. Contudo, o volume de publicações revisadas permitiu atender ao propósito exploratório abrangente esperado neste tipo de síntese^15^^,^^16^^,^^18^. A análise do extenso volume de evidências representou um desafio metodológico. Idealmente, a extração de dados deve ser conduzida por dois ou mais pesquisadores^15^; entretanto, os processos colaborativos adotados contribuíram para minimizar possíveis vieses de extração e categorização dos resultados. 

A crescente aplicabilidade do Sisvan na produção científica pode ter sido impulsionada pela informatização nacional em 2007, que trouxe melhorias operacionais, o que favoreceu sua consolidação e expansão da cobertura. Em 2014, o sistema registrava todos os municípios, embora o maior volume de dados ainda se concentrasse na infância^122^. Também cresceu o interesse acadêmico em torno desse sistema, evidenciado pelo aumento das publicações, sobretudo após 2021. O aumento da insegurança alimentar, agravado pelas crises política e econômica, repercutiu nas condições de vida da população. Sabe-se que esse cenário incide e se reflete no estado de saúde dos indivíduos, colocando-os em risco de diversas formas de má-nutrição^93^^,^^123^.

O incremento de pesquisas ecológicas condiz com a facilidade operacional e o baixo custo associados às informações secundárias, enquanto dados individualizados anonimizados exigem cessão pelo Ministério da Saúde ou acesso ao módulo restrito da plataforma^7^, limitando o uso por pesquisadores. Todavia, ambos os tipos de dados oferecem potencialidades: os individualizados são considerados ideais para inferir sobre fatores associados ou causais de agravos à saúde, porém os ecológicos possibilitam compreensões dos fenômenos em um nível de determinação mais totalizador, ao captarem condicionantes estruturais. Quando combinados em modelos hierárquicos, como observado nos trabalhos revisados, podem proporcionar compreensões relevantes sobre os fenômenos de interesse, sendo esta uma aplicação significativa de informações individualizadas do Sisvan Web^21^^,^^22^^,^^24^. 

Esse sistema pode sinalizar desigualdades sociais por meio de variáveis sociodemográficas^70^^,^^124^, ainda pouco presentes nos estudos agregados, à exceção do sexo. Na ficha do Sisvan, apenas sexo e raça/cor são de preenchimento obrigatório; pertencimento a povos e comunidades tradicionais está disponível desde 2013^5^^,^^30^. Estudos evidenciaram falhas de preenchimento no campo raça/cor^37^^,^^50^^,^^61^^-^^63^^,^^70^^,^^71^, bem como menor cobertura do Sisvan para a população negra^65^ ou possíveis erros classificatórios ou de registro desta variável^54^.

Entretanto, desde 2017, o centralizador nacional da APS é a via preferencial para enviar os dados da vigilância alimentar e nutricional, em detrimento da digitação direta na plataforma Sisvan Web, cuja entrada será descontinuada conforme avançar a implementação da estratégia e-SUS^12^^,^^125^^,^^126^. Essa interface é oportuna para qualificar informações do Sisvan e fortalecer o uso de informações sociodemográficas na compreensão de determinantes sociais da saúde^124^. 

Relativamente aos indicadores nutricionais, uma pesquisa relatou a preferência pelo peso-para-idade para menores de 5 anos, por dispensar a aferição da altura, minimizando erros nas estimativas e abrangendo um maior número de crianças no Sisvan^103^. Para gestantes, o Ministério da Saúde passou a adotar o ganho de peso gestacional como critério diagnóstico, embora o Sisvan Web ainda utilize o índice de massa corporal por semana gestacional para consolidar os dados dessa população^3^. A não obrigatoriedade de preenchimento do campo de peso pré-gestacional no Sisvan pode incorrer em expressiva ausência desse dado^53^. 

Dados provenientes do Programa Bolsa Família foram os mais utilizados, condizentes com o atrelamento histórico das ações de vigilância nutricional a programas governamentais assistencialistas, como o Programa Bolsa Família desde 2004, e a vinculação desse sistema com o Sisvan Web desde o seu lançamento^4^^,^^127^^,^^128^. Em 2020, desobrigaram-se o cumprimento e o registro das condicionalidades em saúde do Programa Bolsa Família, incluindo a avaliação nutricional. Entre 2021 e 2022, esse programa foi substituído pelo Programa Auxílio Brasil, sendo novamente instituído em junho de 2023. Faz-se necessário compreender o impacto dessas mudanças nos indicadores do Sisvan^129^^-^^131^. 

São incipientes os estudos com dados da estratégia e-SUS, possivelmente pela recente interface entre os sistemas, iniciada em 2017^6^^,^^7^^,^^125^. Essa medida fez crescer o número de registros de consumo alimentar inseridos via esse sistema entre 2015 e 2019. Não há evidências sobre indicadores nutricionais, sendo necessário melhor avaliar os efeitos dessa estratégia sobre o desempenho do Sisvan como um todo^12^.

A expansão de delineamentos ecológicos de série temporal foi possibilitada pelo volume de informação regularmente armazenada no Sisvan Web desde seu advento. Percebeu-se o crescente uso de técnicas estatísticas robustas para estimar tendências temporais, a exemplo da metodologia de Prais-Winsten, indicada para essa finalidade por considerar a autocorrelação serial presente em medidas populacionais. Tal característica viola premissas requeridas em modelos tradicionais de regressão, limitando seu uso em análises temporais ao potencialmente induzir interpretações incorretas sobre os fenômenos^132^^-^^134^. Não se recomenda a operação de centralização da variável "ano" para lidar com a autocorrelação, como identificado em um documento^80^^,^^132^. 

O uso da abordagem de Prais-Winsten cresceu em trabalhos nacionais com diversos indicadores de saúde, mortalidade e desempenho dos serviços^135^. A primeira aplicação dessa técnica a dados do Sisvan data de 2020, em avaliação da cobertura do sistema para crianças da região Norte, entre 2008 e 2017^8^ (busca nas bases Lilacs e Pubmed em agosto de 2025 com os termos "prais-winsten" AND "sisvan"). Houve também aplicação dessa metodologia a dados do Sistema de Vigilância de Fatores de Risco e Proteção para Doenças Crônicas por Inquérito Telefônico, outra estratégia de vigilância alimentar e nutricional^136^^-^^138^. 

Além do uso na vigilância epidemiológica, estudos temporais podem evidenciar efeitos de eventos ou políticas públicas. Para isso, dados coletados rotineiramente, como os do Sisvan, são apropriados ao permitirem construir séries longas. A análise de séries interrompidas, considerado o modelo quasi-experimental mais robusto para essa finalidade, ainda incipiente na área da vigilância nutricional, pode avaliar efeitos longitudinais de eventos/intervenções sobre indicadores alimentares e nutricionais^133^^,^^134^^,^^139^.

Pesquisas com recursos de análise espacial e geocodificação salientam ferramentas metodológicas importantes para a epidemiologia. Sistemas de informação geográfica de livre acesso podem facilitar seu uso ao Sisvan, o que contribui para a visualização espacial dos agravos alimentares e nutricionais e conhecimento de territórios prioritários para intervenções e medidas de vigilância.

Esta revisão de escopo possibilitou identificar elementos a serem explorados em futuras pesquisas: (i) estimativas de tendências com dados do Sisvan Web baseadas em métodos padronizados e robustos, para as diversas fases/evento do curso de vida e localizações geográficas; (ii) análises longitudinais que considerem o efeito de eventos/intervenções populacionais sobre os indicadores nutricionais incrementarão a aplicabilidade das informações da vigilância nutricional; (iii) análises espaciais e geocodificação em escalas intramunicipais permitem acompanhar problemas locais e direcionar esforços efetivos; (iv) melhor compreender o efeito da interoperabilidade entre o Sisvan Web e o centralizador nacional da APS e o impacto das mudanças no acompanhamento das condicionalidades do Programa Bolsa Família sobre indicadores do sistema; (v) sistematizar a ausência de informações demográficas nos macrodados, visando informar setores responsáveis pela gestão da plataforma; e (vi) incorporação de modelos hierárquicos aos microdados para investigar determinantes individuais e contextuais dos agravos nutricionais.

Este trabalho apresentou o estado da arte da produção científica e reconheceu o campo de estudos sobre a vigilância nutricional por meio do Sisvan, caracterizando metodologicamente amplo corpo de evidências e identificando o uso das informações desse sistema. Espera-se que os achados contribuam para informar pesquisas futuras e embasar o aperfeiçoamento do Sisvan.

## Data Availability

Os dados extraídos dos documentos selecionados encontram-se armazenados no software Google Sheets (Alphabet Inc., Califórnia, Estados Unidos) e estarão disponíveis mediante solicitação ao autor correspondente.

## Material Suplementar

Tabela Suplementar
